# Diagnostic Accuracy of *PIK3CA* Mutation Detection by Circulating Free DNA in Breast Cancer: A Meta-Analysis of Diagnostic Test Accuracy

**DOI:** 10.1371/journal.pone.0158143

**Published:** 2016-06-23

**Authors:** Yidong Zhou, Changjun Wang, Hanjiang Zhu, Yan Lin, Bo Pan, Xiaohui Zhang, Xin Huang, Qianqian Xu, Yali Xu, Qiang Sun

**Affiliations:** 1 Department of Breast Surgery, Peking Union Medical College Hospital, Beijing, China, 100730; 2 Department of Dermatology, University of California, San Francisco, California, United States of America, 94143–0989; National Cancer Center, JAPAN

## Abstract

Mutation of p110 alpha-catalytic subunit of phosphatidylinositol 3-kinase (*PIK3CA*) has high predictive and prognostic values for breast cancer. Hence, there has been a marked interest in detecting and monitoring *PIK3CA* genotype with non-invasive technique, such as circulating free DNA (cfDNA). However, the diagnostic accuracy of *PIK3CA* genotyping by cfDNA is still a problem of controversy. Here, we conducted the first meta-analysis to evaluate overall diagnostic performance of cfDNA for *PIK3CA* mutation detection. Literature search was performed in Pubmed, Embase and Cochrane Central Register of Controlled Trials databases. Seven cohorts from five studies with 247 patients were included. The pooled sensitivity, specificity, positive and negative likelihood ratio, diagnostic odds ratio and area under summary receiver operating characteristic curve were calculated for accuracy evaluation. The pooled sensitivity and specificity were 0.86 (95% confidence interval [CI] 0.32–0.99) and 0.98 (95% CI 0.86–1.00), respectively; the pooled positive and negative likelihood ratio were 42.8 (95% CI 5.1–356.9) and 0.14 (95% CI 0.02–1.34), respectively; diagnostic odds ratio for evaluating the overall diagnostic performance was 300 (95% CI 8–11867); area under summary receiver operating characteristic curve reached 0.99 (95% CI 0.97–0.99). Subgroup analysis with metastatic breast cancer revealed remarkable improvement in diagnostic performance (sensitivity: 0.86–0.91; specificity: 0.98; diagnostic odds ratio: 300–428). This meta-analysis proved that detecting *PIK3CA* gene mutation by cfDNA has high diagnostic accuracy in breast cancer, especially for metastatic breast cancer. It may serve as a reliable non-invasive assay for detecting and monitoring *PIK3CA* mutation status in order to deliver personalized and precise treatment.

## Introduction

Subunit p110 alpha of phosphatidylinositol 3-kinase (*PIK3CA*) is one of the most commonly mutated oncogenes in breast cancer [1], which presents in more than 20% of HER2-positive tumors [2]. Investigating the clinical utility of *PIK3CA* mutation as a potential biomarker has aroused great interest. It was shown in preclinical models that oncogenic mutants of *PIK3CA* in HER2-positive cell lines led to consistent activation of downstream PI3K/Akt pathway and resistance to trastuzumab and lapatinib [3–5]. For metastatic breast cancer, PI3K pathway activation associated with PTEN loss and/or *PIK3CA* mutation was correlated with poor response to trastuzumab and shortened survival time [6]; Razis *et al*. demonstrated that higher risk of progression was associated with HER2-positive status and the presence of *PIK3CA* mutations [7]. In adjuvant settings, *PIK3CA* mutation showed a strong correlation with reduced disease free survival and overall survival [8, 9]. NeoALLTO trial [10] and a conjoint study of GeparQuattro, GeparQuinto, and GeparSixto [2] both indicated the association of *PIK3CA* mutation and low pathological complete response rate, which serves as a surrogate endpoint for evaluating prognosis.

Due to the predictive and prognostic value of *PIK3CA* mutation in HER2-positive breast cancer, *PIK3CA* genotyping is of great importance for tailoring precise and personalized treatment. Currently, conventional assay for *PIK3CA* genotyping relies on primary or metastatic lesion biopsy, which may lead to severe adverse events, such as pneumothorax and haemorrhagic shock [11]. Because of these potential severe complications and inaccessibility of metastatic lesion, biopsy may not be able to be performed in all cases. Moreover, as *PIK3CA* mutational status in breast cancer was reported to change dramatically between primary tumors and corresponding metastatic [12, 13], sequential biopsy is essential to monitor treatment response and disease progression. However, the invasive procedures of biopsy can often undermine patient compliance. Hence, the utility of less invasive techniques has sparked a great interest.

Circulating free DNA (cfDNA) detection is one of the attractive alternatives for tumor tissue biopsy [14]. It allows identifying molecular subtypes of metastatic diseases and monitoring tumor in real time, which provides potential to predict early treatment response and achieve timely treatment adjustment [15]. Compared to other circulating biomarkers, cfDNA showed a superior sensitivity to metastatic breast cancer (MBC) and a greater dynamic range correlating with changes in tumor burden. However, there was still no consensus on diagnostic accuracy of detecting *PIK3CA* mutation in cfDNA. Board *et al*. reported a 0% sensitivity of *PIK3CA* genotyping with cfDNA [16]. In contrast, study by Dawson *et al*. [17] and the retrospective cohort study by Higgins *et al*. [18] showed the sensitivity up to 100%. As for specificity, the prospective cohort study by Higgins *et al*. [18] had the lowest specificity of 78%, while several other studies reported a specificity of 100% [16, 17, 19].

Thus, we conducted the first meta-analysis to evaluate the diagnostic performance of *PIK3CA* genotyping with cfDNA in breast cancer patients.

## Methods

### Literature search and study selection

The following database were searched for relevant studies: PubMed (from 1946 to Feb 2015), Embase (host: Ovid, from 1947 to Feb. 2015) and Cochrane Central Register of Controlled Trials (CENTRAL, from 2000 to Feb 2015). The medical terms used for search were ‘PI3K’, ‘PIK3CA’ ‘phosphatidylinositol 3-kinase’, ‘Phosphatidylinositol-4,5-bisphosphate 3-kinase’, ‘phosphatidylinositide 3-kinase’, ‘phosphatidylinositol-3-kinase’, ‘PI 3-kinase’, ‘PI-3K’, ‘phosphoinositide-3-kinase’, ‘phosphatidylinositol-4,5-bisphosphate 3-kinase, catalytic subunit alpha’, ‘breast cancer’, ‘breast neoplasm’, ‘cfDNA’, ‘cell free DNA’, ‘ctDNA’, ‘circulating tumor DNA’, ‘tumor free DNA’, ‘ circulating free DNA’, ‘circulating nucleic acid’, ‘plasma DNA’, ‘serum DNA’ and ‘blood DNA’. Article language was limited to English. All the relevant articles listed in the search results were manually screened to ensure the sensitivity of literature search.

The inclusion criteria of this meta-analysis included: 1) studies about detection accuracy of *PIK3CA* mutation by cfDNA in breast cancer patients; 2) studies with raw data that true-positive, false-positive, false-negative and true-negative could be found or calculated; 3) studies with *PIK3CA* mutation analysis of tumor tissue samples; 4) studies with more than 10 patients. The exclusion criteria included: 1) studies with duplicate data reported; 2) studies that were letters, editorials, reviews, comments, conference abstract or case reports.

Two independent reviewers (Y.D. Zhou and C.J. Wang) evaluated eligibility of studies according to the above criteria. Full-text of potentially relevant studies were obtained and reviewed by the same two reviewers. Disagreement was resolved by consensus (Y.D. Zhou, C.J. Wang, and Q. Sun).

### Data extraction and quality assessment

Two reviewers (Y.D. Zhou and C.J Wang) independently extracted data from all eligible studies. With predesigned data extraction forms, following data were collected: 1) Basic characteristics of included studies: name of the first author; year of publication; country. 2) Cohort level characteristic: study design; number of patients; mean/median age; tumor stage; *PIK3CA* mutation detection assay for cfDNA and tumor sample; *PIK3CA* mutation detected in each study. 3) Outcomes: number of true-positive, false-positive, false-negative, true-negative, sensitivity and specificity. If studies include more than one independent cohort, data from each cohort would be collected individually. Subsequently, the two independent authors evaluated the quality of the studies by Quality Assessment of Diagnostic Accuracy Studies-2 (QUADAS-2) [20].

### Statistical analysis

The main outcome measures included pooled estimation of sensitivity (SE), specificity (SP), positive likelihood ratio (PLR), negative likelihood ratio (NLR) and diagnostic odds ratio (DOR). which is a single indicator measure of the overall diagnostic test accuracy [21]. The summary receiver operating characteristic (SROC) curve was estimated by a bivariate mixed-effects regression model. The area under curve (AUC) of the SROC curve was calculated as an alternative global measurement of test performance.

Several graphical tools were adopted for model checking, such as quantile plot of residual-based goodness-of fit; Chi-squared probability plot of squared Mahalanobis distances for assessing the assumption of bivariate normality; spike plot of Cook's distance to check particularly influential observations; scatter plot for checking outliers by standardized predicted random effects. Bivbox plot was used to assess distributional properties of sensitivity versus specificity as well as identify possible outliers. Chiplot was applied to judge whether the paired performance indices are independent.

Between-study heterogeneity was evaluated by Cochran’s Q test (*p* < 0.05 or I^2^ >50%), as well as subgroup and sensitivity analyses performed according to methods described by Deeks *et al*.[22]. Publication bias was evaluated with funnel plot and the Deek’s funnel plot asymmetry test. Clinical utility of *PIK3CA* mutation detection in cfDNA was presented with Fagan’s plot and probability modifying plot.

All the statistical analyses were conducted by Stata software (version 12.0, College Station, TX).

## Results

### Literature selection and general information

**Fig 1:**
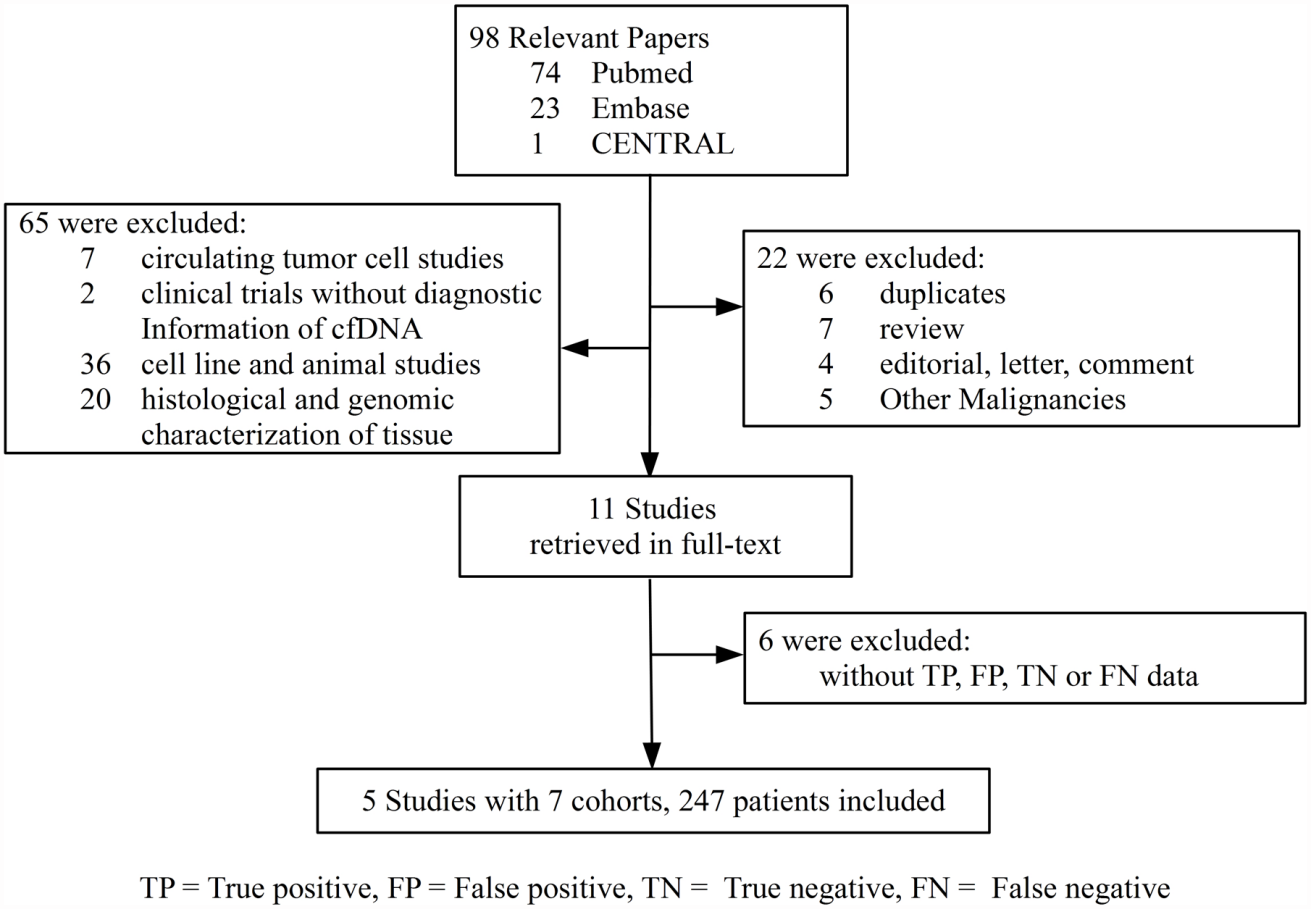
Flowchart of articles reviewed and included in meta-analysis.

Ninety-eight relevant records were found in Pubmed, Embase and CENTRAL databases. After removing obviously irrelevant reports, full-text of 11 articles were retrieved for detailed evaluation. Ultimately, five studies [16–19, 23] with 247 patients were eligible for this meta-analysis (See “S1 Text Excluded full-text articles” for reasons of exclusion). Fig 1 showed the flowchart of literature search and selection. Two selected studies involved two cohorts, respectively [16, 18]. Since there was no overlap between the two cohorts in each study, each cohort was considered to be independent.

The seven cohort studies were composed by six prospective studies and one retrospective study [18]; two cohorts (28.6%) were early breast cancer patients, and the others were metastatic ones. As for the timing of blood and tissue sampling, three cohorts (42.9%) [18, 19, 23] collected the blood and tissue sample synchronously, two cohorts (28.6%) [17, 18] were at different time points, and the two cohorts (28.6%) in the study by Board *et al*. either did not provide relevant information or had a mixing pattern [16]. Heterogeneity existed in tissue source of reference tests (three [42.6%] from primary lesion [16, 17, 19], three [42.6%] from either primary lesion or metastatic lesion [16, 18, 23], and one was unavailable [18]). Regarding to cfDNA testing methods, five cohorts (71.4%) used polymerase chain reaction (PCR) to detect multiple point mutations (p.E545K, p.E542K, p.H1047R and p.H1047L), and the others (28.6%) sequenced the whole *PIK3CA* gene with Next-Generation Sequencing (NGS). Sensitivity and specificity of each cohort ranged from 0.00 to 1.00 and from 0.78 to 1.00, respectively (Table 1). Quality assessment was presented as a bar graph using QUADAS-2 tool (S1 Fig).

**Table 1:** Characteristics of studies included in the meta-analysis of the diagnostic accuracy of *PIK3CA* mutation detection in cfDNA.

No.	Study	Country	Design	N	Age	stage	*PIK3CA* mutation detection assay		*PIK3CA* mutation	TP	FP	FN	TN	SE (95% CI)	SP (95% CI)
							Tumor sample (Source)	cfDNA (Timing: same/different as tumor sampling)							
1	Beaver 2014	US	Pro.	29	Median 60 (38–77)	EBC	PCR (PL)	PCR (Same)	p.E545K p.H1047R	14	0	1	14	0.93 (0.68–1)	1.00 (0.77–1.00)
2	Board(E) 2010*	UK	Pro.	30	Average 64 (39–88)	EBC	PCR (PL)	PCR (NA)	p.E545k p.E542K p.H1047R p.H1047L	0	0	14	16	0.00 (0.00–0.23)	1.00 (0.79–1.00)
3	Board(M) 2010^@^	UK	Pro.	41	Average 59 (43–79)	MBC	PCR (~27% ML; ~73% PL)	PCR (~27% same)	p.E545k p.E542K p.H1047R p.H1047L	8	1	2	30	0.80 (0.44–0.97)	0.97 (0.83–1.00)
4	Dawson 2013	UK	Pro.	30	Median 64 (43–85)	MBC	Tagged-amplicon deep sequencing Paired-end Whole genome sequencing (PL)	PCR, Tagged-amplicon deep sequencing (Different)	PI3K gene	12	0	0	18	1.00 (0.74–1.00)	1.00 (0.81–1.00)
5	Higgins(P) 2012^&^	US	Pro.	51	Median 56 (36–85)	MBC	PCR (NA)	PCR (Different)	p.E545K p.H1047R	8	8	6	29	0.57 (0.29–0.82)	0.78 (0.62–0.90)
6	Higgins(R) 2012^#^	Germany	Retro.	49	Median 62 (39–84)	MBC/EBC	PCR (8% ML, 92%PL)	PCR (Same)	p.E545K, p.H1047R, p.H1047L	14	0	0	35	1.00 (0.77–1.00)	1.00 (0.90–1.00)
7	Rothe 2014	France	Pro.	17	Median 48 (35–62)	MBC	NGS (PL/ ML)	NGS (Same)	PI3K gene	3	1	1	12	0.75 (0.19–0.99)	0.92 (0.64–1.00)

US = United States; UK = United Kingdom; Pro. = Prospective; Retro. = Retrospective; EBC = Early breast cancer; MBC = Metastatic breast cancer; PCR = polymerase chain reaction; NGS = Next-Generation Sequencing; PL = Primary lesion, ML = Metastatic lesion, NA = Not available; TP = True positive; FP = False positive; FN = False negative; TN = True negative; SE = Sensitivity; SP = Specificity; CI = Confidence interval

*Early breast cancer in the study by Board *et al*.^17^;

^@^ Metastatic breast cancer cohort in the study by Board *et al*.^17^;

^&^ the prospective cohort in the study by Higgins *et al*.^18^;

^#^ Retrospective cohort in the study by Higgins *et al*.^18^;

Graphical tools were employed for model checking, outlier identification and detection of possibly influential data. The bivariate mixed-effects regression model was well-fitting for the dataset (S2A Fig), and the cohorts included approximately matched with bivariate normality assumption (S2B Fig). Study by Board *et al*. [16] had strong influence on pooled results (S2C Fig). No outliers were identified by scatter plot (S2D Fig), while the two cohorts in Higgins *et al*.’s study [18] were indicated as outliers by bivbox plot (S3 Fig).

### Diagnostic accuracy of detecting *PIK3CA* mutation in cfDNA

**Fig 2:**
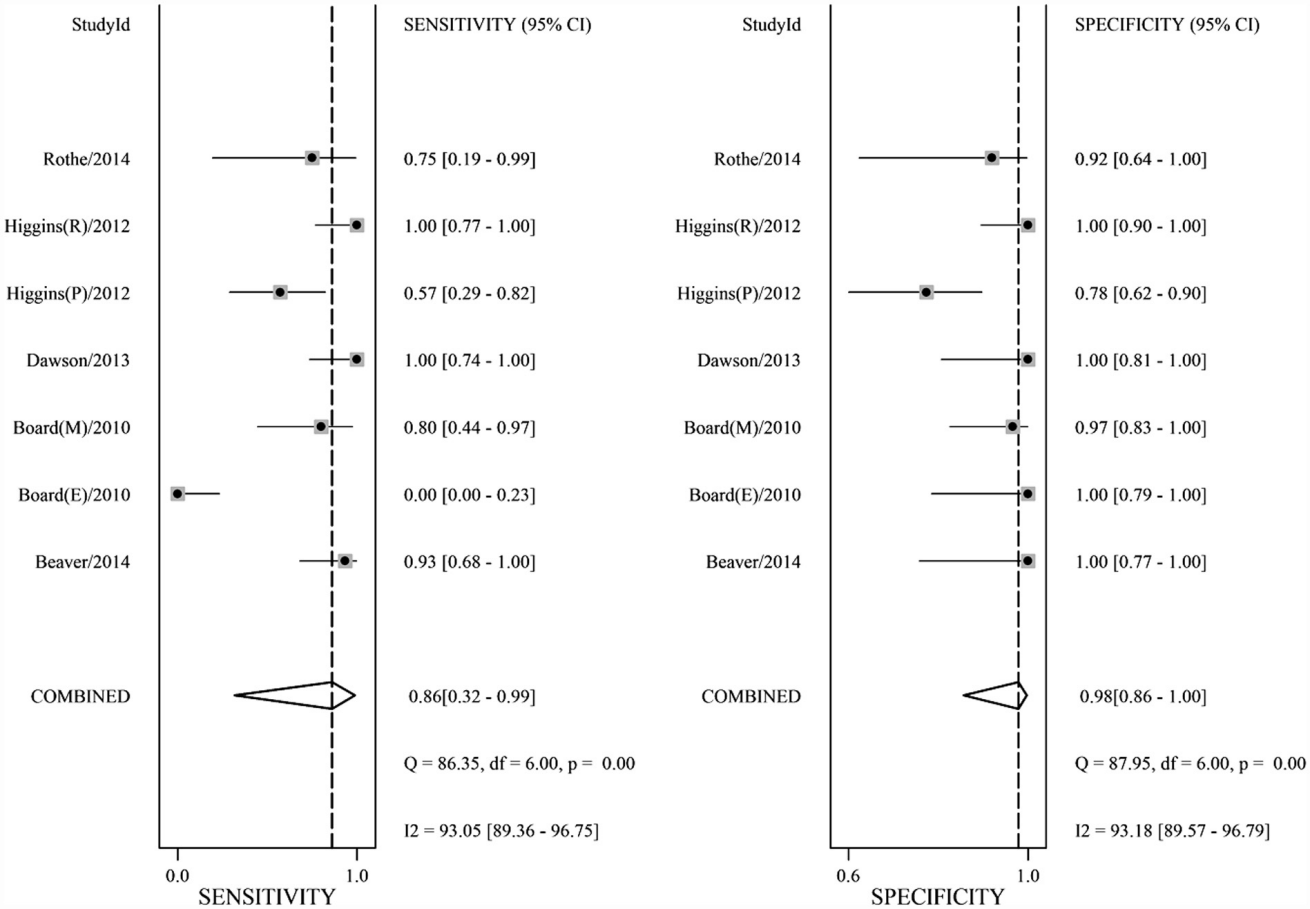
Forest plot showing study-specific and overall sensitivities and specificities with corresponding heterogeneity evaluation.

**Fig 3:**
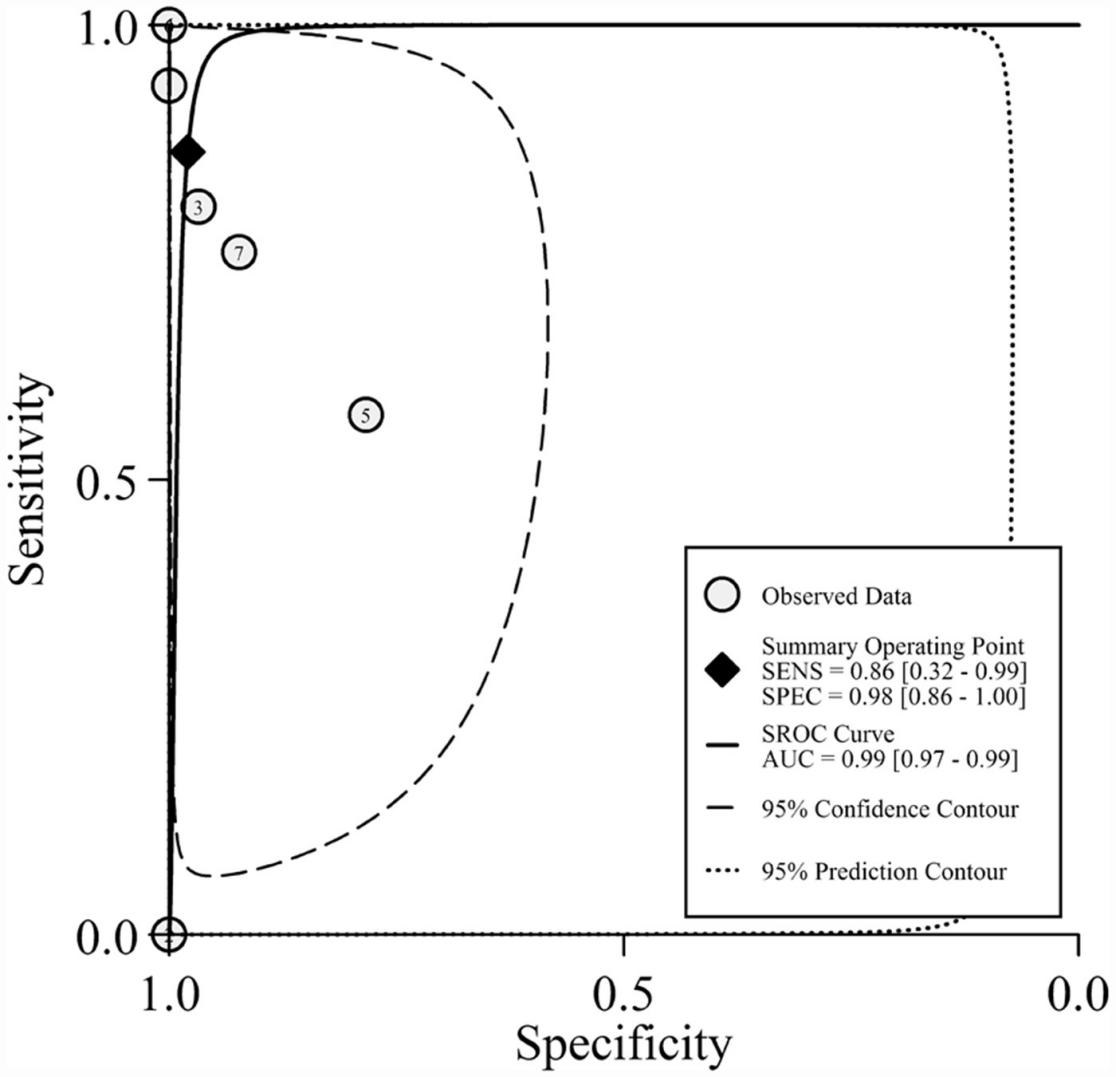
SROC curve with confidence and prediction regions around overall operating sensitivity and specificity point

The pooled SE and SP of *PIK3CA* mutation detection in cfDNA of breast cancer was 0.86 (95% confidence interval [CI] 0.32–0.99) and 0.98 (95% CI 0.86–1.00), respectively; the pooled PLR, NLR were 42.8 (95% CI 5.1–356.9) and 0.14 (95% CI 0.02–1.34), respectively. DOR which generally evaluated the diagnostic test performance reached 300 (95% CI 8–11867). Heterogeneity existed among the included studies (Cochrane’s Q *p* < 0.001, I^2^ 80%) (Fig 2, Table 2). SROC curve (Fig 3) with AUC of 0.99 (95% CI 0.97–0.99) indicated a high diagnostic accuracy. Empirical Bayes forest plot (S4 Fig) presented estimation of the true sensitivity and specificity in each included study.

**Table 2:** Pooled results and subgroup analysis of the meta-analysis for the diagnostic accuracy of *PIK3CA* mutation detection in cfDNA.

Analysis	SE	SP	PLR	NLR	DOR	AUC	Cochrane’s Q	I^2^ (%)
**All studies**	0.86 (0.32, 0.99)	0.98 (0.86, 1.00)	42.8 (5.1, 356.9)	0.14 (0.02, 1.34)	300 (8, 11867)	0.99 (0.97, 0.99)	*p* <0.001	80 (58, 100)
**MBC subgroup**	0.91 (0.58, 0.99)	0.98 (0.78, 1.00)	39.0 (3.2, 475.5)	0.09 (0.01, 0.59)	428 (8, 23007)	0.99 (0.97, 0.99)	*p* = 0.487	0 (0, 100)
**Prospective design**	0.75 (0.22, 0.97)	0.97 (0.83, 0.99)	22.9 (3.5, 149.1)	0.26 (0.04, 1.51)	89 (4, 1984)	0.99 (0.97, 0.99)	*p* = 0.003	81 (59, 100)
Without* **outliers**	0.78 (0.13, 0.99)	0.98 (0.92, 0.99)	36.1 (7.9, 164.7)	0.22 (0.02, 2.61)	164 (5, 4980)	0.99 (0.97, 0.99)	*p* = 0.008	76 (47, 100)

* Outliers were defined by bivbox plot.

Numbers in parentheses are 95% confidence intervals. SE = Sensitivity; SP = Specificity; PLR = positive likelihood ratio; NLR = negative likelihood ratio; DOR = Diagnostic odds ratio; CI = Confidence interval;

### Heterogeneity investigation

**Fig 4:**
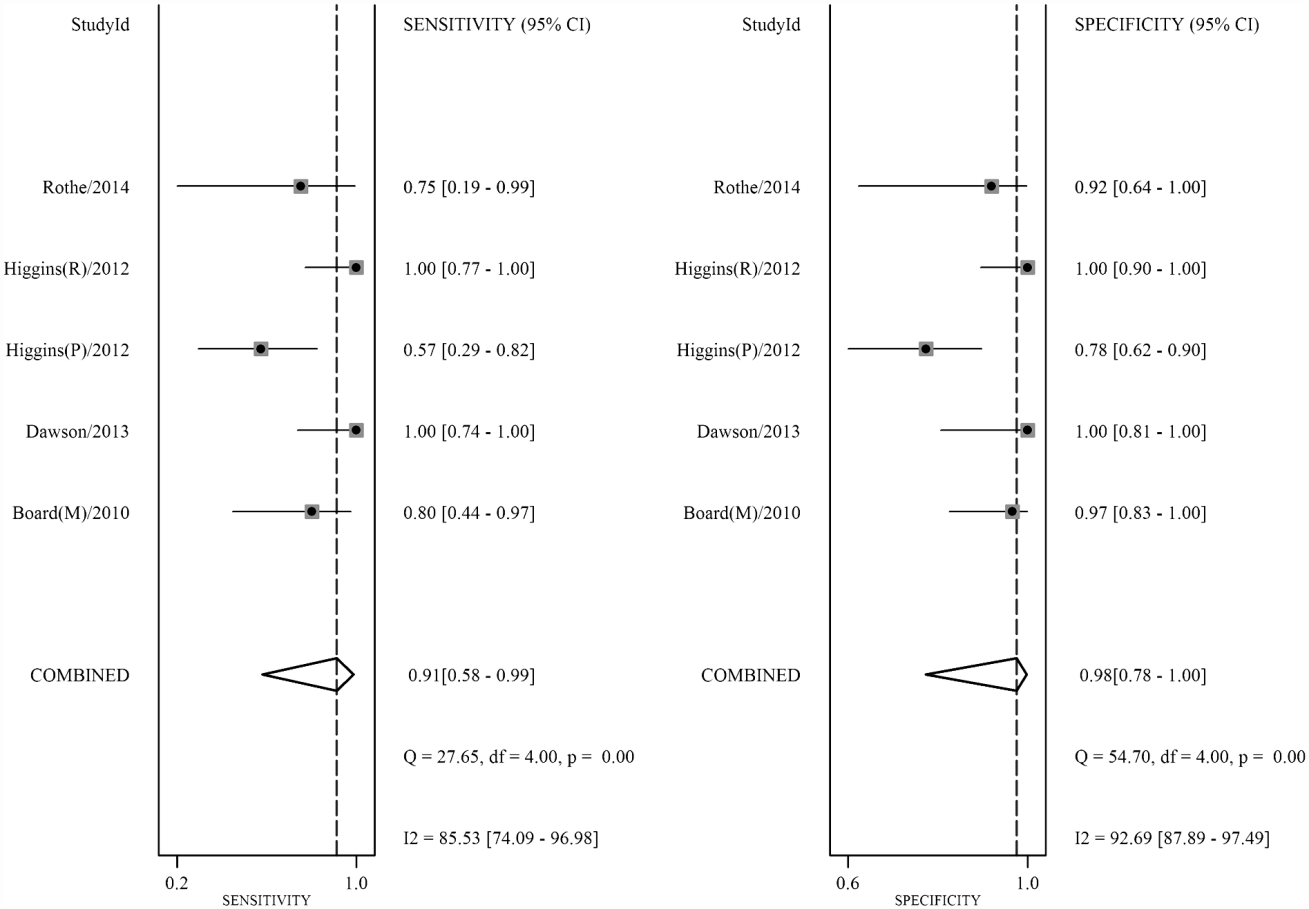
Forest plot showing study-specific and overall sensitivities and specificities with corresponding heterogeneity evaluation of MBC subgroup.

**Fig 5:**
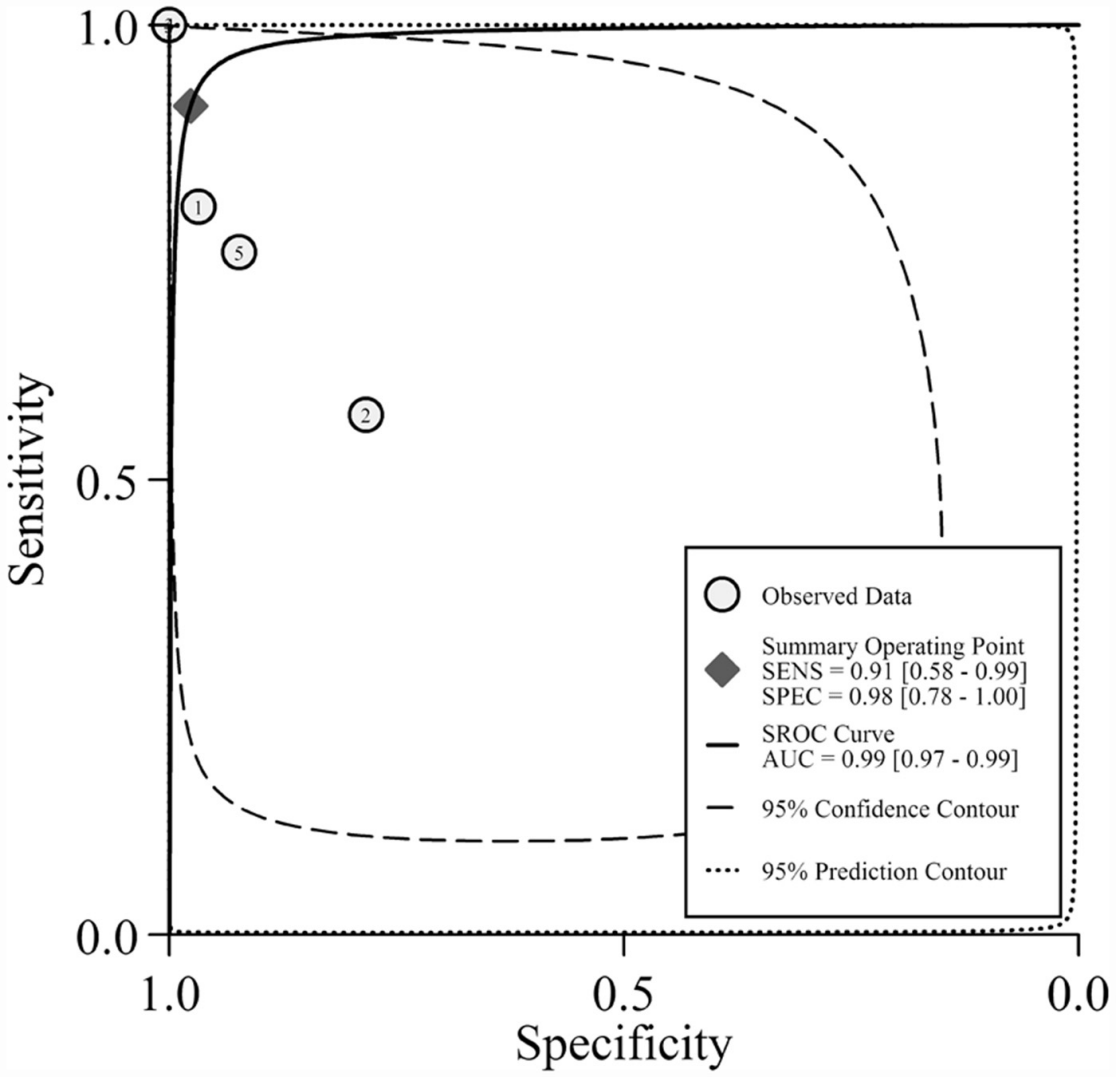
SROC curve with confidence and prediction regions around overall operating sensitivity and specificity point of MBC subgroup.

Heterogeneity investigation was performed according to different covariates, such as tumor stage, study design and whether outlier or not. For tumor stage, I^2^ dropped from 80% to 0% in cohorts of MBC patients, indicating that all MBC cohorts were homogeneous. Forest plot and SROC curve of MBC subgroups were presented in Figs 4 and 5. Accordingly, the diagnostic accuracy in MBC patients was improved (SE from 0.86 to 0.91, SP remains 0.98, and DOR from 300 to 428, Table 2). These results indicate that detecting *PIK3CA* in cfDNA was highly consistent and more accurate in MBC patients.

**Fig 6:**
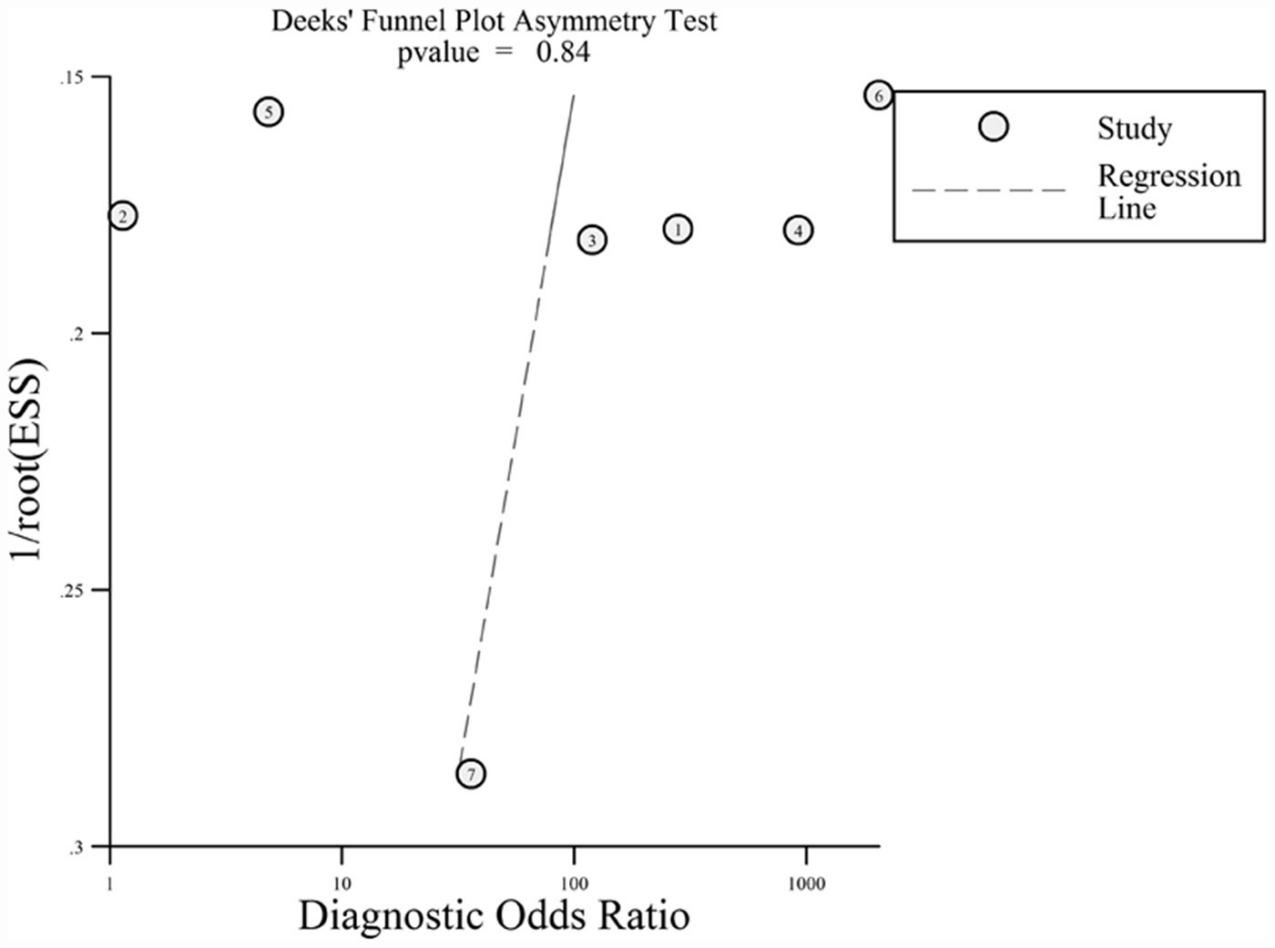
Funnel plot with superimposed regression line.

Excluding the retrospective study and outliers [18] resulted in no significant improvement in heterogeneity (I^2^ decreased from 81% to 76%, details of diagnostic accuracy shown in Table 2). No significant publication bias was determined Deek’s funnel plot asymmetry test (*p* = 0.84, Fig 6).

### Clinical utility

**Fig 7:**
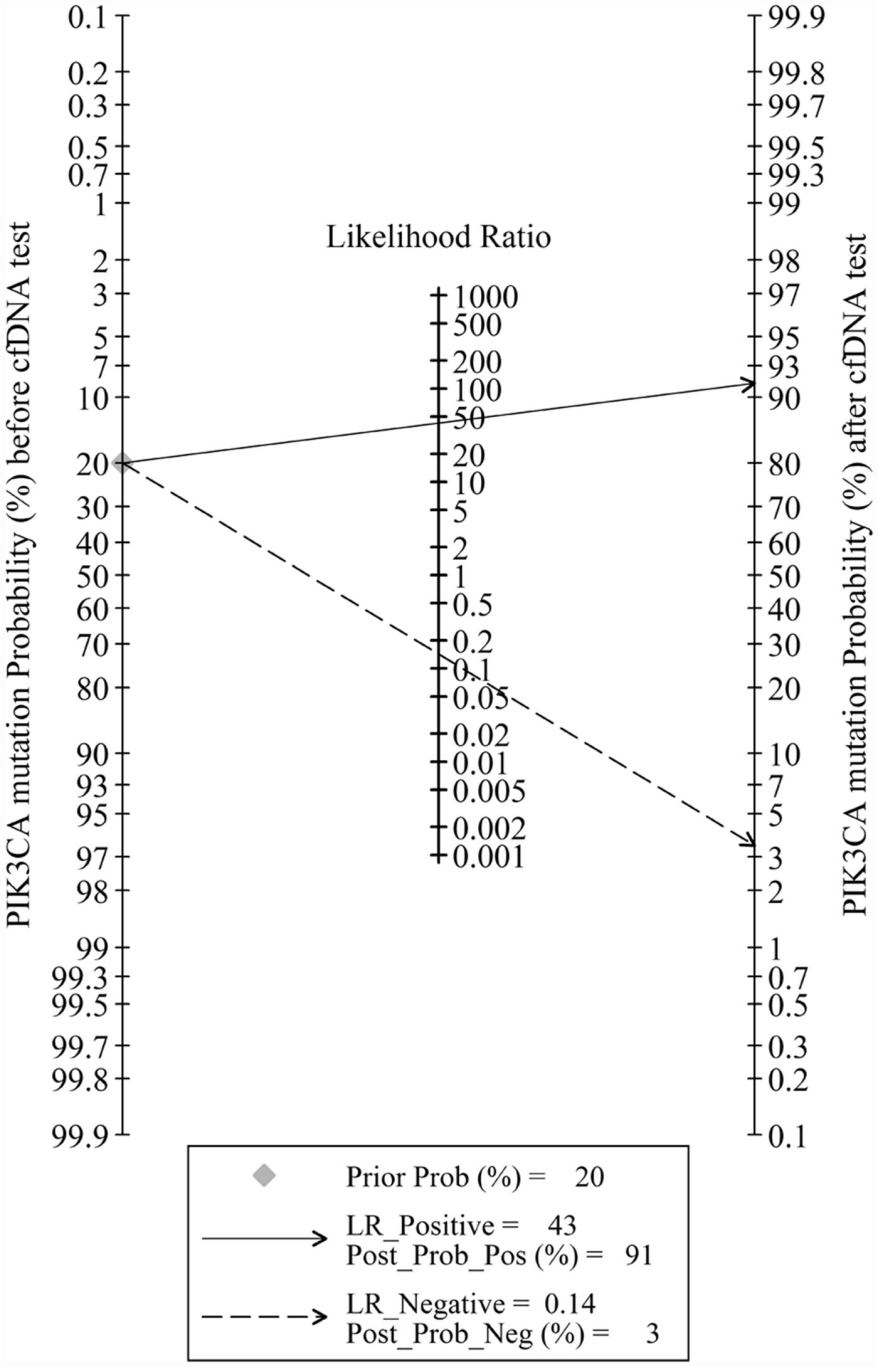
Fagan plot analysis to evaluate the clinical utility of *PIK3CA* mutation detection in cfDNA.

**Fig 8:**
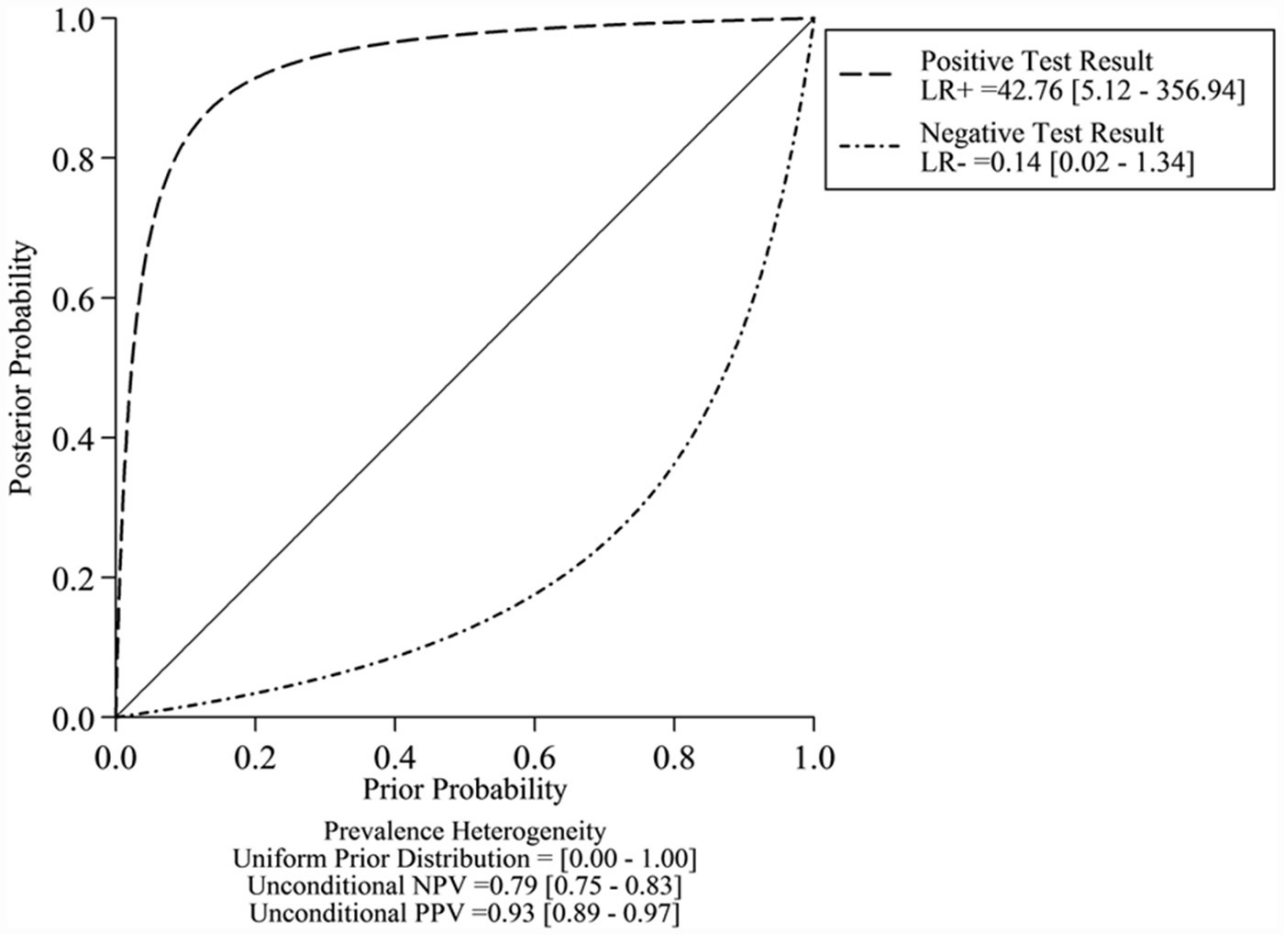
Probability Modifying Plot.

Fagan’s plot revealed a dramatic improvement of post-test probability (Fig 7). When pre-test probability of *PIK3CA* mutation was set to 20%, using cfDNA as a source to detect *PIK3CA* mutation could significantly raise the post-test probability of positive result to 91%, and lower the post-test probability of negative result to 3%. Probability modifying plot with predictive values is showed in Fig 8.

## Discussion

CfDNA provides a less invasive and more convenient assay for monitoring *PIK3CA* genotype. Janku *et al*. determined concordance between mutation analysis of tumor tissue and that of plasma cfDNA on various advanced cancers, including colorectal cancer, melanoma, non-small cell lung cancer, appendiceal cancer, ovarian cancer and uterine cancer. Results showed high sensitivity and specificity of *PIK3CA* mutation detection with cfDNA (0.86 and 0.91, respectively) [24]. Similarly, the concordance rates between tumor tissue sample and cfDNA for detecting *PIK3CA* exon 9/20 mutations were as high as 97%-100% in lung cancer [25]. However, the diagnostic accuracy of plasma cfDNA remains controversial in breast cancer. Studies reported sensitivity and specificity of *PIK3CA* mutation detection in cfDNA ranging from 0 to 100% and 78 to 100%, respectively [16–19, 23]. Therefore, we systematically reviewed studies on breast cancer to explore the diagnostic accuracy of detecting *PIK3CA* mutation in cfDNA.

High values of pooled sensitivity, specificity and DOR indicated a high diagnostic accuracy of plasma cfDNA for prediction of *PIK3CA* mutation. AUC, serving as an overall summary index of test performance, is considered as an indicator of good diagnostic performance when the value is greater than 0.90 [26]. Therefore, the calculated value of 0.99 in our study suggested an excellent diagnostic accuracy of cfDNA. DOR for overall result and metastatic subgroup were both up to 300, suggesting good discriminatory performance of cfDNA test. But it should be noted that the confidence interval of DOR were fairly large (Table 2) due to small number of studies included in this meta-anlysis, the pooled results should be applied with caution that DOR may have great variance resulting in less precise estimates.

Since AUC and DOR are not easy to interpret and apply in clinical practice [27], likelihood ratios were calculated as more clinically meaningful indicators [28]. Usually, PLR >10.0 or NLR <0.1 was regarded to be sufficient to generate large and conclusive improvement from pre-test to post-test probability. In this study, pooled PLR reached 42.8, meaning that patients with positive cfDNA result have more than 40 fold higher odds to have *PIK3CA* mutation in tumor sample compared to healthy controls. The pooled NLR of 0.14 suggests that patients with negative *PIK3CA* mutation in cfDNA still have 14% possibility to have *PIK3CA* mutation in their tumor sample. Hence, although a negative result could not exclude the possibility of *PIK3CA* mutation in primary or metastatic lesions, the diagnosis could be confirmed on patients with a positive result of *PIK3CA* mutation in cfDNA. In another word, *PIK3CA* mutation detection in cfDNA may not serve as a screening test, but it qualified as a confirmative assay.

Studies on melanoma and colorectal cancer demonstrated that cfDNA mutation detection had a stage-dependent effect; patients with early stage cancer had a lower detection rate for mutation in cfDNA, compared to those with advanced disease [29, 30]; tumor DNA was prone to present in the circulation of late stage disease than that of early stage [31]. Therefore, we conducted the subgroup analysis for MBC cohorts to determine whether *PIK3CA* mutation detection in cfDNA is also stage-dependent. In MBC subgroup, except for a slight decrease in PLR (from 42.8 to 39.0), all the parameters for evaluating cfDNA diagnostic performance had remarkable improvements (SE from 0.86 to 0.91, SP remained 0.98, NLR from 0.14 to 0.09, DOR from 300 to 428). Given that PLR was larger than 10 and NLR was less than 0.10 in MBC subgroup, a conclusion could be drawn that the diagnostic accuracy of detecting *PIK3CA* mutation in cfDNA for MBC was high enough to serve as both confirmative and exclusive assay. Moreover, at the exclusion of early breast cancer cohorts, the inter-study heterogeneity within MBC subgroups was eliminated (I^2^ from 80% to 0%, Cochrane’s Q *p* value from <0.001 to 0.487), which revealed strong homegeneity among MBC cohorts and further validated our results.

Although no heterogeneity was determined in MBC subgroup, some confounding factors could potentially influence the accuracy of pooled results. Studies by Dawson *et al*. and Higgins *et al*. collected blood samples when disease recurred and compared the *PIK3CA* genotype in cfDNA with that in primary tumor, instead of metastatic lesion [17, 18]. This could probably raise false negative and false positive rates, since recent reports validated that *PIK3CA* mutational status in breast cancer differed approximately 18% of the time between primary tumors and corresponding metastatic disease with changes in both directions (wild type to mutant type, and vice versa) [12, 13]. Besides, it was proven that multiple genetically diverse colonial subpopulations exist within primary breast cancers. According to previously accepted models of tumor progression and metastatic dissemination punctuated by colonial expansions [32], the incongruity of *PIK3CA* genotype between primary and metastatic lesions could also compromise the diagnostic performance of *PIK3CA* mutation detection in cfDNA.

Different assays have been used for detecting *PIK3CA* mutation in cfDNA. Angulo *et al*. reported that PCR has lower limit of detection than NGS in EGFR mutation detection for lung cancer, meaning higher sensitivity of PCR [33]. Due to the relative small amount of cfDNA in blood, NGS may not be able to detect the presence of *PIK3CA* mutation and result in false negative results [23]. Hence, inclusion of studies using NGS as detection method could reduce the sensitivity of the pooled results. However, NGS showed several advantages. For example, it could screen multiple mutations for multiple genes simultaneously [34], provide enormous information on novel mutations, and serve as a better option for mutation screening [34, 35]. At present, it could be a reasonable strategy to screen multiple genomic mutations in tissue sample by NGS, and monitor the change of mutations in cfDNA by PCR for follow-up.

This study has several limitations. First, meta-regression and subgroup analysis on several covariates were unable to perform, such as early breast cancer subgroup and subgroup taking blood and tumor sample concurrently. Second, the present study failed to include patient survival information, therefore the prognostic and predictive values of *PIK3CA* mutation in cfDNA were difficult to evaluate. Moreover, grey literature was not included in this meta-analysis. As grey literature trials usually showed an overall worse treatment effect than published trials [36], our study had the potential risk to overestimate the accuracy of *PKI3CA* mutation detection in cfDNA.

## Conclusion

In conclusion, our meta-analysis supports the notion that detecting *PIK3CA* gene mutation in cfDNA has high diagnostic value in breast cancer patients, especially for MBC. It could probably serve as a reliable non-invasive assay for detecting *PIK3CA* mutation and monitoring *PIK3CA* genotype changes after treatments to guide personalized therapy. Further large-scale studies are required to confirm our findings and differentiate the optimal patient subgroup that is suitable for using this assay as routine clinical practice. Additionally, the validation of the prognostic power of cfDNA in breast cancer should be conducted by large multicenter prospective clinical trials.

## Supporting Information

S1 Fig Overall quality assessments of included studies (QUADAS-2 tool). (TIF)

S2 Fig Graphical depiction of residual-based goodness-of-fit (A), bivariate normality (B), influence and outlier detection analysis (C and D, respectively). (TIF)

S3 Fig Bivbox Plot. (TIF)

S4 Fig Paired forest plot depiction of empirical Bayes predicted versus observed sensitivity and specificity. (TIF)

S1 Text Excluded full-text articles. (DOCX)

S2 Text Appendix PRISMA Checklist. (DOCX)
