# Supplementary figures and images for: Diagnostic Accuracy of PIK3CA Mutation Detection by Circulating Free DNA in Breast Cancer: A Meta-Analysis of Diagnostic Test Accuracy
Source: PLoS One. 2016 Jun 23;11(6):e0158143. doi: 10.1371/journal.pone.0158143 (PMC4918940; doi:10.1371/journal.pone.0158143)

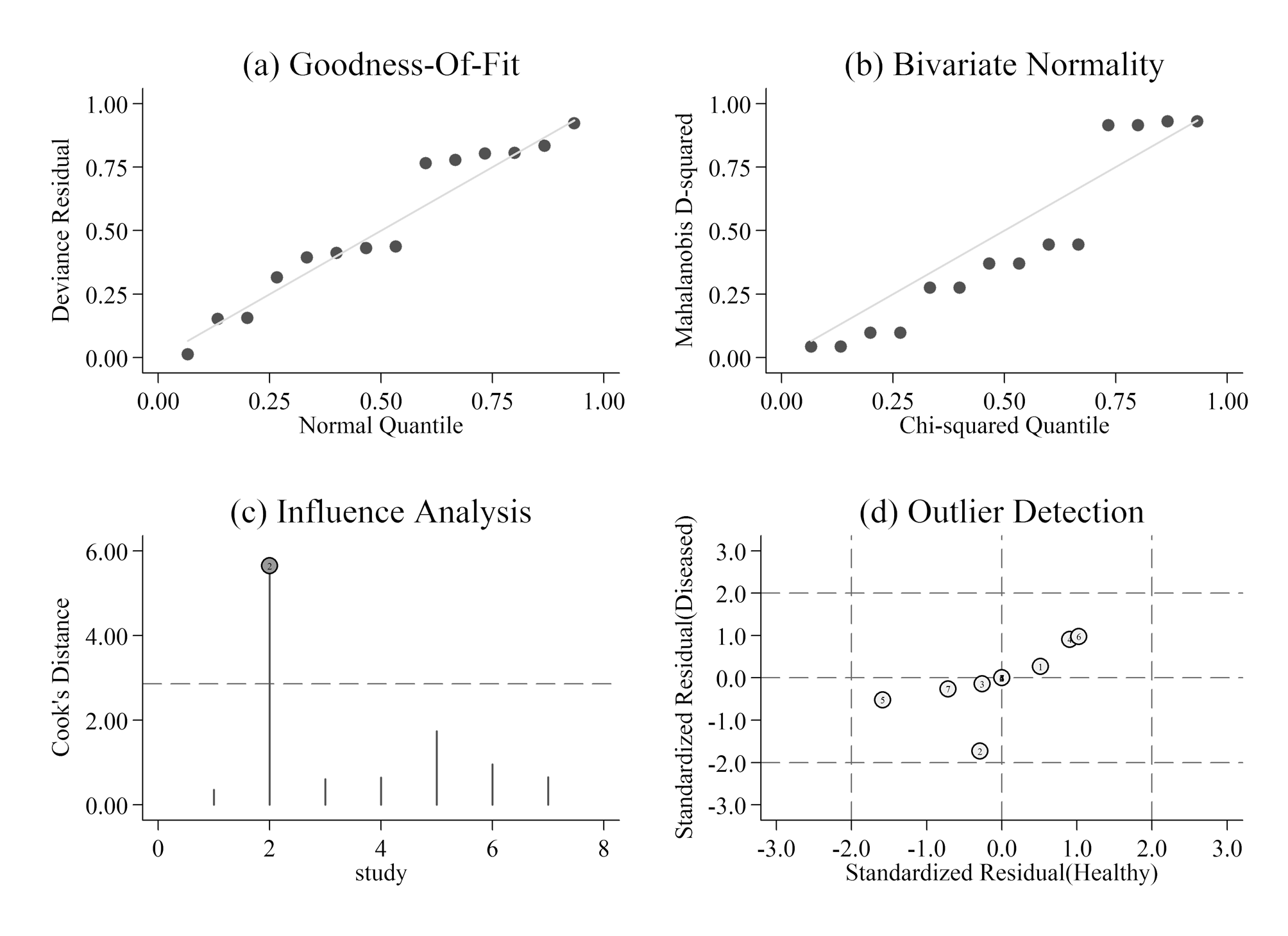

Supplement: S2 Fig — (TIF) [file pone.0158143.s002.tif]

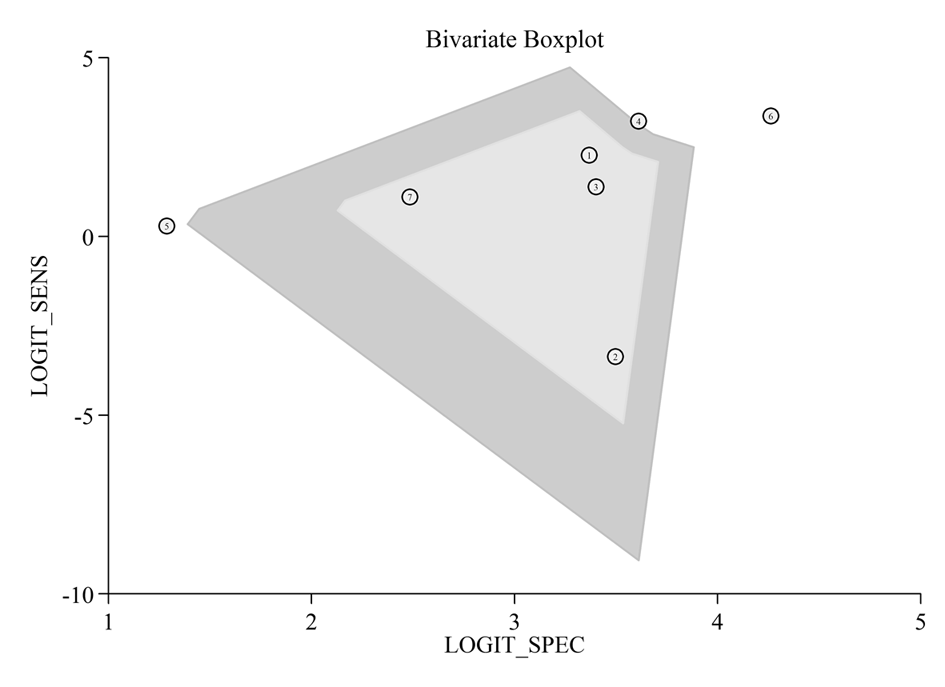

Supplement: S3 Fig — (TIF) [file pone.0158143.s003.tif]

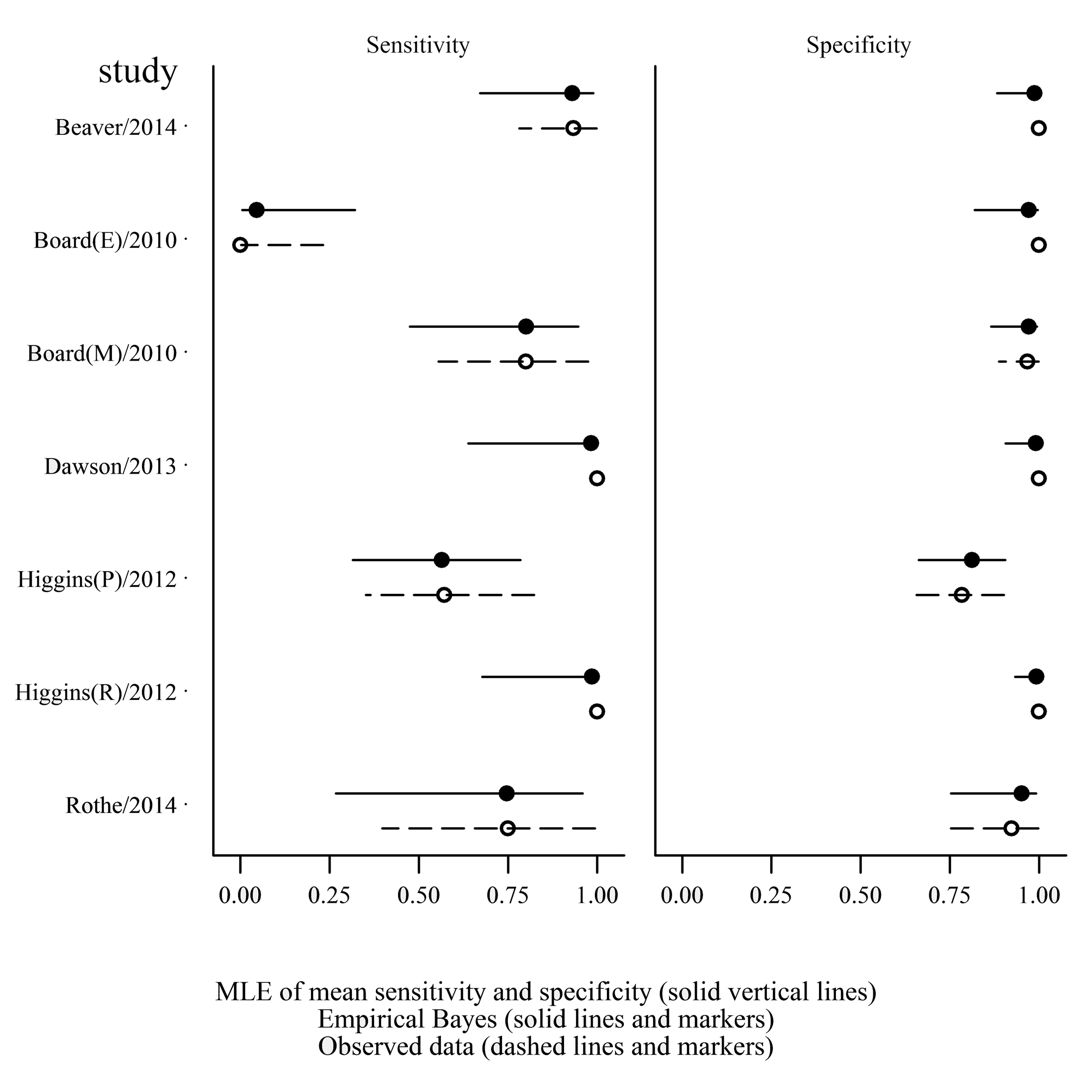

Supplement: S4 Fig — (TIF) [file pone.0158143.s004.tif]
