# Supplementary material for: Diagnostic Accuracy of PIK3CA Mutation Detection by Circulating Free DNA in Breast Cancer: A Meta-Analysis of Diagnostic Test Accuracy
Source: PLoS One. 2016 Jun 23;11(6):e0158143. doi: 10.1371/journal.pone.0158143 (PMC4918940; doi:10.1371/journal.pone.0158143)
Supplement: S1 Text — (DOCX) [file pone.0158143.s005.docx]

- The Six Full-text Articles Excluded

1. Murtaza M, Dawson SJ, Tsui DW, Gale D, Forshew T, Piskorz AM, et al. Non-invasive analysis of acquired resistance to cancer therapy by sequencing of plasma DNA. Nature. 2013;497(7447):108-12. Epub 2013/04/09. doi: 10.1038/nature12065. PubMed PMID: 23563269.

**Reasons for exclusion**: This study has only one breast cancer patient with *PIK3CA* mutation, so we were unable to extract FP, TP, FN and TN data for meta-analysis.

2. Ramirez-Ardila DE, Helmijr JC, Look MP, Lurkin I, Ruigrok-Ritstier K, van Laere S, et al. Hotspot mutations in PIK3CA associate with first-line treatment outcome for aromatase inhibitors but not for tamoxifen. Breast cancer research and treatment. 2013;139(1):39-49. Epub 2013/04/18. doi: 10.1007/s10549-013-2529-7. PubMed PMID: 23592373.

**Reasons for exclusion**: This study was excluded due to lack of *PIK3CA* mutation data for cfDNA, so FP, TP, FN and TN data are unavailable.

3. Maruyama N, Miyoshi Y, Taguchi T, Tamaki Y, Monden M, Noguchi S. Clinicopathologic analysis of breast cancers with PIK3CA mutations in Japanese women. Clinical cancer research : an official journal of the American Association for Cancer Research. 2007;13(2 Pt 1):408-14. Epub 2007/01/05. doi: 10.1158/1078-0432.ccr-06-0267. PubMed PMID: 17202311.

**Reasons for exclusion**: This study did not evaluate *PIK3CA* mutation in cfDNA, so FP, TP, FN and TN data are unavailable.

4. Kalinsky K, Jacks LM, Heguy A, Patil S, Drobnjak M, Bhanot UK, et al. PIK3CA mutation associates with improved outcome in breast cancer. Clinical cancer research : an official journal of the American Association for Cancer Research. 2009;15(16):5049-59. Epub 2009/08/13. doi: 10.1158/1078-0432.ccr-09-0632. PubMed PMID: 19671852.

**Reasons for exclusion**: This study was excluded due to lack of *PIK3CA* mutation data for cfDNA, so FP, TP, FN and TN data are unavailable.

5. Cizkova M, Susini A, Vacher S, Cizeron-Clairac G, Andrieu C, Driouch K, et al. PIK3CA mutation impact on survival in breast cancer patients and in ERalpha, PR and ERBB2-based subgroups. Breast cancer research : BCR. 2012;14(1):R28. Epub 2012/02/15. doi: 10.1186/bcr3113. PubMed PMID: 22330809; PubMed Central PMCID: PMCPMC3496146.

**Reasons for exclusion**: This study did not evaluate *PIK3CA* mutation in cfDNA, so FP, TP, FN and TN data are unavailable.

6. Cizkova M, Dujaric ME, Lehmann-Che J, Scott V, Tembo O, Asselain B, et al. Outcome impact of PIK3CA mutations in HER2-positive breast cancer patients treated with trastuzumab. British journal of cancer. 2013;108(9):1807-9. Epub 2013/04/25. doi: 10.1038/bjc.2013.164. PubMed PMID: 23612454; PubMed Central PMCID: PMCPMC3658522.

**Reasons for exclusion**: This study was excluded since it did not report *PIK3CA* mutation in cfDNA.
